# The 1-h fraud detection challenge

**DOI:** 10.1007/s00210-021-02120-3

**Published:** 2021-07-10

**Authors:** Marcel A. G. van der Heyden

**Affiliations:** 1 Department of Medical Physiology, Division of Heart & Lungs, University Medical Center Utrecht, Yalelaan 50, 3584 CM Utrecht, The Netherlands; 2 Graduate School of Life Sciences, Utrecht University, Utrecht, The Netherlands

**Keywords:** Fraud, Paper mill, Misconduct, Education, Fabrication, Falsification

## Abstract

Publications baring falsified and fabricated images appear frequently in the primary literature. Industrialized forms of image forgery as practiced by the so-called paper mills worsen the current situation even further. Good education and awareness within the scientific society are essential to create an environment in which honesty and trust are the prime values in experimental research. Here I focus on the detection of publication fraud and provide some examples and advice. Finally, my views on the future of fraud detection and prevention are given.

## Introduction

Publication fraud is a plague that spreads among scientific journals. I define publication fraud as the action to produce scientific publications that have the intention to mislead the reader. The most extreme variant within this spectrum is publication of falsified or fabricated data. Today, these practices are not only the result of individual scientific cheaters, but is scaled up in the so-called “paper mills,” companies whose products are papers full of falsified and fabricated data (Else and Van Noorden 2021). I became interested in publication fraud almost 15 years ago during the aftermath of the Korean stem cell fraud (Saunders and Savulescu 2008; Van der Heyden et al. 2009). It was in the 2005 Science paper in which the authors provided panels having partly overlapping immunofluorescent images of identical colonies depicted as independent clones, that struck me (Couzin 2006). After my awareness was raised, I encountered more frequently examples of publication fraud, in many fields of the life sciences, both in scientific publications and at conferences. Many eye-catching cases were featured in the layman media (Table 1), and thereby, these acts of misconduct shake public trust in the scientific process also. Since I am passionate about the profession of experimental science as such, I could not stand publications that deliberately go against the honesty and trust that form the pillars of the research métier. Furthermore, the European Conduct of Science denotes that “Ignoring putative violations of research integrity by others…” is not acceptable for anyone working in research (ALLEA 2017). For these reasons, I informed editors of the affected journals a number of times on “figure issues” as I tend to call them. Of course, I always use my full name and affiliations and mention absence of conflicts of interest with the signaled publications. Except from a few dissonant replies in the early days, most journal editors and publishers reacted very positive to such information. Confronted with the sheer amount of, and increase in publication fraud I encountered over the last 10 years, and also filed by many different blogs like RetractionWatch and PubPeer (Table 2), it appeared to me that the problem could not be solved any more by detecting and reporting “figure issues.” Although I have to stress that this guarding and cleaning of the existing scientific literature is of invaluable importance. However, when we want to stop the production of falsified and fabricated material, the scientific community has to take their responsibility to prevent publication fraud (Korte and Van der Heyden 2017). One of the cornerstones to this end is good education of all involved in scientific research. Therefore, I developed lectures and workshops on publication fraud, using real life examples taken from recent publications, and often specifically selected for the research area in which the audience is active. Many eyes were opened and often people were genuinely shocked about the fact that such obvious falsified data could have been published, and felt the urge that this could not be accepted in our profession. One of the items during the lectures is the so-called “1-h fraud detection challenge.” Here I state that anyone can find falsified or fabricated material in papers published the week before the lecture. I ask the participants to take the challenge, and guide them with some practical advice on how to approach this issue and provide examples. And of course, to keep it a challenge, the journal under inspection should be peer reviewed and carrying an impact factor.

**Table 1:** Examples of fraud cases, including image forgery, covered in the layman media

Case	Country of origin	Unmasked^a^	Research field	Type of data^b^
Marion Brach	Germany	1997	Hematology	Northern blot
Woo-Suk Hwang	South-Korea	2005	Stem cells	Histology
Luk van Parijs	USA	2005	Immunology	Western blot, flow cytometry
Jon Sudbø	Norway	2006	Cancer biology	Histology
Dipak Das	USA	2012	Cardiovascular	Western blot
Haruko Obokata	Japan	2014	Stem cells	DNA gel, histology
Piero Anversa	USA	2015	Stem cells	Western blot, histology
Oona Lönnstedt	Sweden	2017	Marine biology	Specimen photographs

^a^In many cases, suspicions have been raised in the field earlier

^b^This summary is not intended to be comprehensive

**Table 2:** Web resources discussing publication fraud, including image forgery

Name + year	Character	Activity	Weblink
Copy, Shake, and Paste 2006	Germany based blog on plagiarism and scientific misconduct	Journalism news-blog	https://copy-shake-paste.blogspot.com
Riddled 2009	New-Zealand based blog including potential publication fraud identification	Post-publication peer review	http://eusa-riddled.blogspot.com
RetractionWatch 2010	US based non-profit news forum reporting on paper retractions providing insight in underlying mechanisms	Journalism news-blog	https://retractionwatch.com
PubPeer 2012	US based non-profit online journal club	Post-publication peer review	https://pubpeer.com
Forbetterscience 2015	Germany based science journalism on scientific misconduct in all forms	Journalism news-blog	https://forbetterscience.com
Science integrity digest 2019	US based blog on scientific integrity by Elisabeth Bik	Journalism news-blog	https://scienceintegritydigest.com

## How to detect “figure issues” yourself within 1 h?

First, one has to be aware that data falsification and fabrication is occurring frequently, although exact numbers are difficult to provide and may vary between 0.3 and 4.7% of all published primary research (e.g., Thiese et al. 2017). But these numbers may be even higher. Using an automatic image manipulation detection setup, Bucci (2018) found that approximately 6% of the papers investigated contained manipulated images, whereas another study found that approximately 23% of a set of papers from basic oncology contained data duplications (Oksvold 2016). In my experience, new thrilling research fields attracting many readers increase the chance of finding “figure issues.” This was the case in the early years of human stem cell research, later in the field of micro- and other non-coding RNAs, and in general in every research field with a fancy prefix, like currently nano-. The still ongoing the COVID-19 crisis already yielded many publications in this field, which on several occasions resulted in retractions and scandals (Boetto et al 2020).

**Fig. 1:**
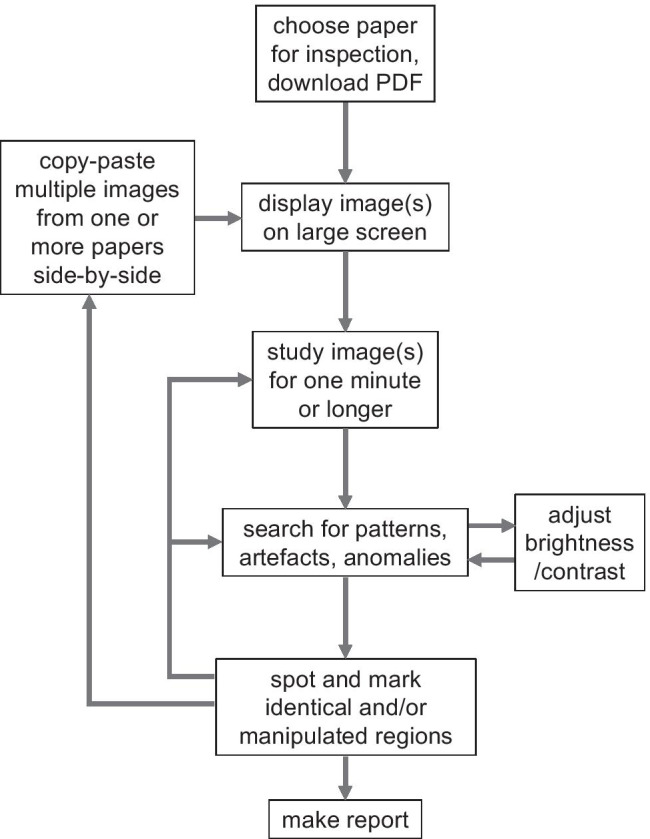
Flow diagram depicting subsequent steps and loops used in image fraud detection endeavors

**Fig. 2:**
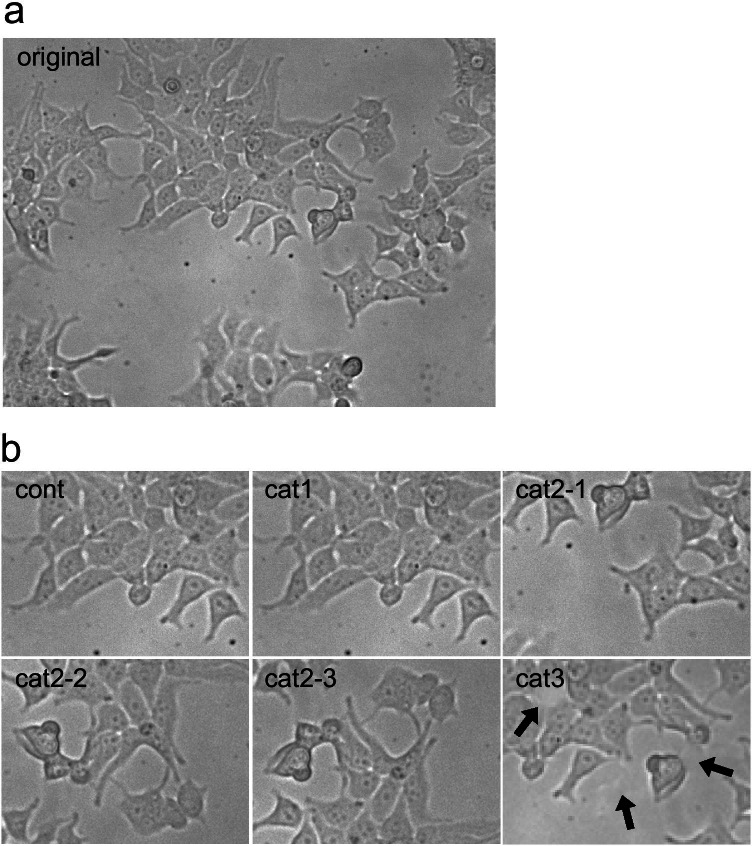
Examples of falsification categories. **a** Original phase-contrast image of HEK293 cells (20 × magnification). **b** Selected parts of panel **a**. cont, non-manipulated part of panel **a**. cat1, copy-paste from cont. cat2-1, different selection from panel **a**. cat2-2 different and rotated selection from panel **a**. cat2-3 horizontally flipped image from cat2-2. cat3 different selection from panel **a** in which three cells (arrows) are erased by the Photoshop cloning tool. Figure 1 was produced in less than 30 min, including microscopical imaging, using the Photoshop and PowerPoint software

Secondly, select a journal and start looking at the research papers published last week, just by opening the PDFs of the issue (Fig. 1). It certainly helps when using a big screen, or even better, multiple big screens. After opening the PDF, start looking at figures first, without becoming influenced by the accompanying text in which authors will guide the reader through the data, since this may affect ones visual perception. Knowledge of the underlying experimental techniques is helpful, and in a later stage when potential publication fraud is noted, even essential. Look for patterns in the images when comparing multiple panels or figures. When looking at the sky at night, you may recognize constellations, or at least interesting figures like the Plough/Big Dipper, which you immediately identify next days when looking up. In this respect, experimental artifacts like air bubbles in western blots or spots in histochemistry are most helpful. Figure 2 provides a first practice. Panel A displays an original phase contrast microscopical recording of HEK293 cells. In panel B, I display parts of the original recording, with or without falsification or fabrication. The panel labelled as “Cont” is the control situation, “cat1” is a copy-paste of “cont.” Bik et al. (2016) categorized such copy paste images as category 1, which can result from genuine error. “Cat2-1,” “cat2-2,” and “cat2-3” are parts of panel A in which a different part of the original recording is depicted (and “hided” behind the label), rotated, or rotated and mirrored, respectively, which provide clear examples of category 2 manipulations. That is, the author had to put specific efforts within the software to produce these image panels. Panel “Cat3” is an image in which category 1 and 3 are combined. Category 3 means alteration within the image. In this case, I removed three cells by using the “cloning” tool of the Photoshop software (arrows).

**Fig. 3:**
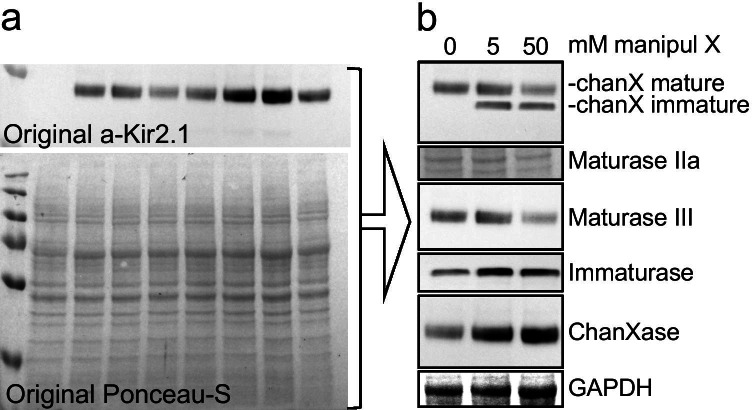
Example of a fabricated western blot image. **a** Top: original western blot of Kir2.1 protein detection on samples from transfected HEK293 cells (lane 1, marker; lane 2, non-transfected cells), bottom, total protein staining of the original western blot by Ponceau-S. **b** Reconstructed western blot from the original recordings from panel **a**. A fake legend may be read as: “*manipul X dose-dependent decreases mature chanX protein by decreasing Maturase III and increasing Immaturase and ChanXase expression.*” Panel **b** was produced in less than 25 min from the original material presented in **a** using the PowerPoint software

Figure 3 provides an example of western blot manipulation. Western blots are notorious difficult to judge, since very often genuine bands indeed look very similar. In this case, however, I took one western blot on which a number of protein samples were run, after which the Kir2.1 protein was detected. Total protein staining on the western blot was performed using Ponceau-S. From these two blots presented in panel A, I subsequently constructed panel B using a combination of category 1, 2, and 3 manipulations. For example, the “GAPDH” was constructed using a small part of the Ponceau-S recording, in which I simply altered brightness and contrast. Obviously, this results in an “equal loading” signal. Lane 2 and 3 of “Immaturase” and “ChanXase” are the same recordings, however vertically narrowed, rotated and mirrored in combination with altered brightness and contrast.

**Fig. 4:**
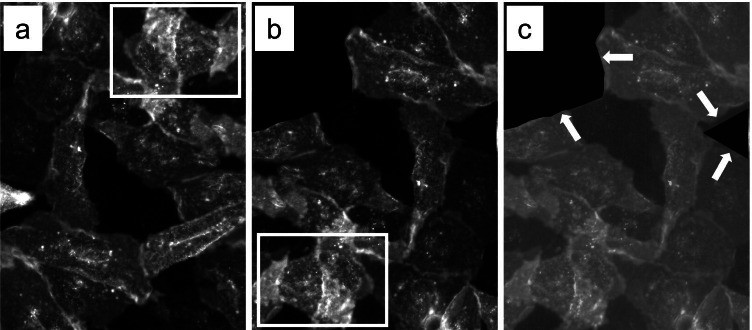
Example of a fabricated immunohistochemistry figure and its obvious detection using altered brightness/contrast. **a** Original image of Kir2.1 labelled CHO cells. **b** Category 2 and 3 manipulation from panel **a** by 180° rotation and covering two areas by black shapes. **c** Altered contrast uncovers the black covering shapes (arrows). White boxes in **a** and **b** outline identical areas for easy recognition. Panel **b** was produced in 35 min from the original material presented in **a** using the Photoshop and PowerPoint software

In cases of complex manipulation, it may help to copy-paste the image in PowerPoint, or similar software, and depict the identified falsified/fabricated parts by circles, boxes, etc. (Fig. 4A, B). This will provide overview, after which you can further look in the non-marked parts of the figure. Furthermore, once the image is put in such software, one can easily alter brightness and contrast, which sometimes uncovers manipulation scars, like boxes within an image. Figure 4C provides an example. Panel A is the original immunofluorescent microscopy image, panel B shows the manipulated image I constructed, whereas panel C displays the same image as in B, but now with altered brightness. Arrows indicate the manipulation scars. When a potential falsified/fabricated image is noticed, one has to read the accompanying text to make sure that your initial view is indeed correct, and does not result from an unfamiliar experimental setup or technique. Then, it may be worthwhile to screen other papers from the same author group. In my experience, publication fraud is certainly not always an incident within a research group, and similar techniques of falsification and fabrication are being used in previous or subsequent publications. Furthermore, this will also identify deliberate re-use of identical data or the so-called “stock images” (Byrne and Christopher 2020) without mention, in subsequent publication or even complete double publications. By performing these additional screens, it also became clear to me that acts of publication fraud can move with one or more authors to their new positions at subsequent research institutes. Moreover, such additional screening can lead to identification of large clusters of publication fraud.

## A few words of caution

Do not get cynical as the far majority of published work is the result of honest efforts (e.g., Bik et al. 2016). Furthermore, the process of experimental research is complicated, uses often newly developed techniques, and has to deal with inherent biological variability. Therefore, we have to recognize that errors will unfortunately be made, but this is not publication fraud. When accusations of publication fraud are made, one should be absolutely sure using compelling evidence. Without that, you may, and most likely will, harm colleagues and careers inappropriately.

## The future of publication fraud detection

The abovementioned workflow, although yielding results, is very labor intensive. Recently, paper mills have received much attention in the scientific press (Table 3). Many journals receive paper mill manuscript, some as many of 5–10% of their total amount of submissions. This vast amount of submissions may require resilient response. Some publishers appoint specific “spotters” whose task it is to detect “figure issues” in incoming manuscripts (Else and Van Noorden 2021). Also, image analysis software is being developed and several publishers are currently implementing these in their submission portals, as many of them already did in an earlier stage for plagiarism detection software (Pomputius 2019; Else and Van Noorden 2021). Without doubt, this will result in interception of falsified and fabricated data. On the other hand, techniques for producing fraudulent images are improving also and even artificial intelligence approaches are being used that create western blot images that cannot be distinguished from genuine experimental results (Byrne and Christopher 2020; Else and Van Noorden 2021). Commercial parties, as the previously mentioned paper mills, have strong interests in these developments. As such, the fraud production and detection arm race appear to have started. Likely, there will be no definitive winner in the end.

**Table 3:** PubMed indexed papers on the subject paper mills in scientific publishing

Author, year	Type	Content
Hvistendahl, 2013	News	An early disconcerting report on the scientific publication industry in China, including a description of practices (“…the company buys data from a national laboratory in Hunan province.”) that maybe describes how the paper mill industry produce manuscripts
Byrne et al., 2019	Review	Building on their 2017 published discovery on a large cluster of similar papers, the authors state that understudied human-genes form an easy target for the paper mill industry. This generates large amounts of false data that may pose serious delays in genuine biomarker research. The authors sensibly hypothesize on the modus operandi of paper mills, which also provides options for preventing publication of paper mill products
Byrne and Christopher, 2020	Review	A comprehensive review on paper mills, their history, business model, and presumed operational methods. It introduces the terms “invented images” and “stock images,” and provides methods for screening paper mill products by editors, journal staff, and peer reviewers. Includes several citations to interesting non-PubMed indexed papers on publication pressure
Moore, 2020	Editorial	Argues that unfindable scientific content of predatory journal papers and preprint servers feed the paper mill industry. Plagiarism detection software is fooled and image manipulation detection by the human eye still forms the cornerstone in uncovering paper mill products
Hackett and Kelly, 2020	Editorial	States that journals, like *BiO* are victim of the paper mill industry, and defines their strategy (Publishing Ethics Coordinator, in house detection by image spotters, software development, raw data requests upon identification of image issues) to defend against paper mill products
Teixeira da Silva, 2021	Letter	Argues that besides paper mills and their customers, also reviewers (publons), editors (citations), journals (impact factor), and indexing agencies and search machines benefit from paper mill activities. Upon discovery of a paper mill (paper), all in the publication ecosystem that profit should suffer consequences
Mallapaty, 2020	News	Reports on new rules from the Chinese science ministry on dealing with research misconduct. These new rules also target those active in the paper mill industry
Frederickson and Herzog, 2021	Editorial	Indicates that paper mills have affected the *Molecular Therapy* journal family, and states new submission requirements to fight against paper mill products entering their journals
Seifert, 2021	Editorial	Indicates that *Naunyn–Schmiedeberg’s Archives of Pharmacology* became a victim of paper mills. Lists 20 features of paper mill products, and provides strategies (institutional email address requirement, supplemental original source data, supplemental immunoblot data, explicit author statement that no paper mill was involved) to prevent paper mill submissions
Heck et al., 2021	Editorial	Summarizes the hallmarks of paper mill products. Reports that 5–10% of total amount of recent submission to the *International Journal of Cancer* bear such suspicious marks. Warns the paper mill industry and their costumers not to submit their papers to this journal since their money will be lost
Else and Van Noorden, 2021	Comment	Reports on the act of transparency by the *Royal Society of Chemistry* on a large series of retractions of paper mill products from their journals. Describes the paper mill industry characteristics and the work of research integrity analysts, also known as “research integrity sleuths.”

## What can be done?

There are many factors that stimulate the occurrence of publication fraud. All types of incentives, as for example, publication based job promotions, are adding to the publication fraud epidemic. In my opinion, publications presenting the outcome of scientific research, the so called “originals,” must be solely used for their prime purpose that is archiving and exchange of scientific results within the community of researchers, and nothing else. We can see steps taken in these directions resulting in initiatives like the San Francisco Declaration on Research Assessment (DORA) (2013) and Science in Transition (Dijstelbloem et al. 2013; Benedictus et al. 2016) that state that evaluation of researchers should be based on multiple indexes, one of which is scientific content of a paper, and not the impact factor of the journal in which it is published. However, many steps still need to be taken (McKiernan et al. 2019; Jacobs 2021). Secondly, fabricated and falsified images and other data have difficulties to stand up against requests for providing the original underlying raw data. Therefore, providing original data, as for example, western blots that form the basis of an edited (and readable) figure panel, as many journals currently require (e.g., Frederickson & Herzog 2021; Seifert 2021), will certainly prevent publication fraud to some extent at the moment. At least until artificial intelligence blots are being produced in large quantities. Thirdly, global, uniform, and well defined training programs in research integrity for all that are active in the field of science is essential (Steneck 2013; Kalichman 2013 and 2014). The European Code of Conduct for Research Integrity, put forward by ALLEA that consists of 59 scientific (national) academies across Europe, states that associated research institutions and organizations “develop appropriate and adequate training in ethics and research integrity” (ALLEA 2017). Indeed, many universities have PhD programs on research integrity, including defining and detecting research misconduct (Abdi et al. 2021). Furthermore, the quality of such programs is still improving (Watts et al. 2017). Today, many free online resources are available and easily accessible for responsible conduct of research (RCR) education, but with a strong focus on the field of Life Sciences (Pizzolato et al. 2020). These important efforts in RCR education will hopefully yield generations of scientists that value the intrinsic importance of science above ordinary temptations. When we reach that point in time, fraud detection will become what it should be: a despairing challenge.
